# Endothelin‐1 response to whole‐body vibration in obese and normal weight individuals

**DOI:** 10.14814/phy2.15335

**Published:** 2022-05-20

**Authors:** Adeola A. Sanni‐Ajibaye, Anson M. Blanks, Cassandra C. Derella, Abigayle B. Simon, Paula Rodriguez‐Miguelez, Jacob Looney, Jinhee Jeong, Jeffrey Thomas, David W. Stepp, Neal L. Weintraub, Xiaoling Wang, Ryan A. Harris

**Affiliations:** ^1^ Department of Medicine Georgia Prevention Institute Augusta University Augusta Georgia USA; ^2^ Department of Kinesiology and Health Sciences Virginia Commonwealth University Richmond Virginia USA; ^3^ Vascular Biology Center Augusta University Augusta Georgia USA; ^4^ Sport and Exercise Science Research Institute Ulster University Jordanstown Northern Ireland United Kingdom

**Keywords:** adiposity, endothelin‐1, exercise, whole‐body vibration

## Abstract

Upregulation of endothelin‐1 (ET‐1) is the hallmark of various cardiovascular diseases (CVD). The purpose of the present study was to assess the ET‐1 response to an acute bout of whole‐body vibration (WBV) in humans and to determine the role of adiposity. Twenty‐two participants volunteered for the study; they were grouped into overweight/obese [(OW/OB): *n* = 11, Age: 33 ± 4 years, Body mass index (BMI): 35 ± 10 kg/m^2^] or normal weight [(NW): *n* = 11, Age: 28 ± 7 years, BMI: 21 ± 2 kg/m^2^]. Participants engaged in 10 cycles of WBV exercise (1 cycle = 1 min WBV followed by 30 s of rest). Blood samples were analyzed for ET‐1 pre‐WBV (PRE), immediately post (POST), 1 h (1H), 3 h (3H), and 24 h (24H) post‐WBV. There was a significant time main effect of WBV on circulating ET‐1 (*F* = 12.5, *p* < 0.001); however, the ET‐1 response was similar (*F* = 0.180, *p* = 0.677) between groups. Specifically, compared to PRE, a significant increase in ET‐1 was observed at 1H (*p* = 0.017) and 3H (*p* = 0.025). In addition, concentrations of ET‐1 were significantly lower at 24H compared to PRE (*p* = 0.019), 1H (*p* < 0.001), and 3H (*p* < 0.001). Maximal oxygen uptake during WBV was similar between the two groups. Acute WBV resulted in an initial rise in ET‐1, followed by a significantly lower ET‐1 at 24H in both groups. Findings support the utility of routine WBV exercise to elicit a decrease in ET‐1 and improve CVD risk, similar to what has been reported with traditional modes of exercise.

## Graphical abstract

The study demonstrates that 10 cycles of 1 min of vibration exercise (10 min total) followed by 30 s of standing rest results in a reduction in circulating ET‐1 after 24 h of exercise in Normal weight and Overweight/Obese participants.

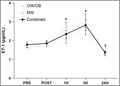

## 1 INTRODUCTION

Cardiovascular disease (CVD) is the leading cause of death in industrialized nations, costing ~ $863 billion in medical care (Fox et al., 2016; Lovren et al., 2015). Upregulation of endothelin‐1 (ET‐1) is the hallmark of various pathologies including CVD. ET‐1 is a potent vasoconstricting peptide that is released by the endothelium and plays a critical role in the regulation of vascular tone. In addition, increased concentrations of ET‐1 contributes to endothelial dysfunction, hypertension, (Cardillo et al., 2000; Weil et al., 2011; Williams et al., 2002), a decline in cardiopulmonary function, and an overall increased risk of CVD; particularly in obese individuals (Serés et al., 2003). In fact, ET‐1 concentrations are elevated in individuals with greater adiposity (Ferri et al., 1995). Accordingly, strategies that improve CVD risk through targeting a reduction in circulating concentrations of ET‐1 are certainly warranted.

One of the most effective methods of reducing concentrations of ET‐1 and improving overall CVD risk is regular exercise (Agarwal, 2012). Exercise facilitates an increase in ET‐1 into circulation (Maeda et al., 2002). Subsequently, ET‐1 binds to its receptors on the vascular smooth muscle, (Yanagisawa et al., 1988) which contributes to a redistribution of blood flow during exercise (Maeda et al., 2002). A single bout of exercise can cause a transient increase in ET‐1 (Dow et al., 2017); however, chronic exercise has been used to reduce ET‐1‐mediated vasoconstrictor tone in sedentary overweight/obese adults (Maeda et al., 2003), independent of weight loss (Dow et al., 2017).

Despite the evidence supporting the use of regular exercise to reduce ET‐1, many individuals do not adhere to current exercise recommendations, thus further increasing the risk of CVD (Pietilainen et al., 2008). Several barriers have been reported that are preventing traditional exercise initiation and maintenance, particularly in people with increased adiposity (Okifuji & Hare, 2015). Whole‐body vibration (WBV) has emerged as a more tolerable, low‐impact exercise mimetic (Zago et al., 2018) and may represent an alternative to traditional forms of exercise. In fact, among others, beneficial responses to WBV such as improved strength and reduced vascular stiffness have been reported in sedentary healthy adults (Dolny & Reyes, 2008), older adults (Merriman & Jackson, 2009), and even people with multiple sclerosis (Jackson et al., 2008).

Although the effects of WBV on CVD risk has yet to be elucidated, recent findings from our lab demonstrate that WBV elicits a differential immune, metabolic, and myokine response in obese and normal weight individuals (Blanks et al., 2020). The ET‐1 response to WBV exercise in humans is unknown. Accordingly, the purpose of this study was to assess the ET‐1 response to an acute bout of WBV in humans and determine if the response is different between normal weight and overweight/obese individuals. We hypothesized that (1) acute WBV would cause a transient increase in ET‐1 that was followed by a reduction in circulating ET‐1, and (2) the response would be more beneficial in overweight/obese individuals; findings that are similar to what has been previously reported with traditional modes of exercise.

## 2 METHODS

### 2.1 Participants

Twenty‐two apparently healthy women and men ages 18–45 years volunteered to take part in this study. Participants were excluded if they were active smokers or had recently quit within the previous 6 months, had a clinical diagnosis of cardiovascular disease, hypertension, metabolic disease, or were taking any vasoactive medications (i.e., nitrates, beta‐blockers, ACE inhibitors, etc.). To examine the effects of adiposity, body mass index (BMI) was used to group participants into either overweight/obese (OW/OB: *n* = 11, BMI ≥ 25 kg/m^2^) or normal weight groups (NW: *n* = 11, BMI < 25 kg/m^2^). The study conforms with the recent declaration of Helsinki and all study protocols were approved by the Institutional Review Board at Augusta University (IRB# 611204).

### 2.2 Experimental design

All participants reported to the Laboratory of Integrative Vascular and Exercise Physiology (LIVEP) at the Georgia Prevention Institute of Augusta University for a preliminary visit that consisted of the informed consent process, anthropometric measures, and body composition assessment. For the experimental visit, participants reported to the LIVEP in the morning following an overnight fast and having abstained from moderate‐to‐vigorous physical activity for 24 h before investigation. Blood samples were collected before (PRE), immediately post (POST), 1‐h post (1H), 3‐h post (3H), and 24‐h post (24H) WBV. Cardiopulmonary and hemodynamic measures were collected throughout the WBV exercise.

### 2.3 Participant characteristics and clinical laboratory values

Height and weight were determined using a stadiometer and standard platform scale (CN20, DETECTO©) and used for calculations of body mass index (BMI). Total body fat, fat mass, and fat‐free mass were determined using dual‐energy X‐ray absorptiometry (QDR‐4500 W; Hologic), and resting systolic and diastolic pressures were evaluated using established protocols (Kapuku et al., 1999). Resting oxygen saturation was obtained using an Onyx II fingertip sensor (Nonin Medical). An intravenous catheter was inserted into an antecubital vein and a 10 mL blood sample was obtained. Fasting concentrations of total cholesterol (TC), high‐density lipoproteins (HDL), low‐density lipoproteins (LDL), triglycerides (TG), and glucose were obtained using a Cholestech LDX point of care analyzer (Alere Inc.). Hemoglobin and hematocrit were determined using a HemoPoint H2 analyzer (Stanbio Laboratories).

### 2.4 Whole‐body vibration

An oscillating side alternating whole‐body vibration platform was used for the study (RS3000, Rock Solid Wholesale). Participants were instructed to remove any footwear and stand mid‐center on the platform with a loose grip on the front rails. Vibration frequency was set to 14 Hz as this frequency has been demonstrated to elicit muscle activation but is well below the frequency in which potential harmful side effects may occur (Cardinale & Wakeling, 2005). The vibration amplitude was set to 2.5 mm. These settings yielded a peak acceleration of 20.19 m/s (≈2.1 g). The protocol consisted of 10 cycles of 1 min of vibration exercise followed by 30 s of standing rest. During the vibration portion of the protocol, participants were instructed to stand in a static squat position, consisting of knee flexion (~60°) with a stable non‐flexed trunk.

### 2.5 Cardiopulmonary and hemodynamic assessments

Measures of cardiopulmonary variables were taken to assess cardiorespiratory load during WBV. Expired gases were collected breath by breath using a TruOne 2400 metabolic cart (Parvo Medics), and the average of the final 30 s of each WBV cycle was used to obtain oxygen consumption (VO_2_) and ventilatory efficiency (VE/VCO_2_ slope). Relative oxygen consumption is presented when normalized to either body mass or fat‐free mass (FFM) in kilograms (kg). Indices of cardiovascular hemodynamics (heart rate [HR], stroke volume [SV], cardiac output [CO], contractility index [CTI], end‐diastolic volume [EDV], and estimated ejection fraction [%EF]) were collected throughout the WBV protocol using a transthoracic impedance cardiography technique (Physioflow^®^; Manatec Biomedical). Physioflow^®^ is both a valid and reproducible assessment of cardiovascular hemodynamic measures in comparison to the invasive thermodilution Swan‐Ganz catheter technique (Charloux et al., 2000).

### 2.6 Circulating concentrations of endothelin‐1

Blood samples were collected at the aforementioned time points and separated via centrifugation. Plasma was isolated and aliquoted, flash‐frozen in liquid nitrogen, and stored at −80 °C until analysis. Plasma concentrations of endothelin‐1 (ET‐1), within the detection range of 0.250–1000 pg/mL, were determined in duplicate using Simple Plex cartridges run on the Ella platform (ProteinSimple) according to the manufacturer's instructions. Any coefficients of variation greater than 20% were repeated.

### 2.7 Statistical analyses

All analyses were performed using SPSS version 25 (IBM Corporation). Repeated‐measures ANOVA was used to test for ET‐1 response to WBV over time. After significance was determined by ANOVA, simple contrasts of within‐subjects were performed to assess the difference between each time point and the PRE value.

Independent samples *t*‐tests were performed to identify differences in demographics, clinical laboratory markers, cardiopulmonary measures, and hemodynamic measures between the two groups. Independent samples *t*‐tests were performed to identify group differences in cardiopulmonary measures following WBV. Values are presented as mean ± standard deviation (SD) unless otherwise stated. An alpha <0.05 was considered statistically significant for all analyses.

## 3 RESULTS

### 3.1 Participant demographic characteristics and clinical laboratory values

Twenty‐two participants completed the study. Participant demographics and clinical laboratory values are presented in Table 1. Significant differences in BMI, body fat percentage, weight, blood lipids, and glucose were observed between groups (all values *p* < 0.05). No significant differences in the remaining characteristics and lab values between groups were observed.

**TABLE 1:** Participant characteristics and laboratory values.

Variables	Overall	Normal weight (NW) *n* = 11	Overweight/Obese (OW/OB) *n* = 11	*p*‐value
Sex (M/W)	12/10	6/5	6/5	
Age (years)	30 ± 6	28 ± 7	33 ± 4	0.040*
Weight (kg)	83 ± 26	62 ± 9	104 ± 20	< 0.001*
Height (cm)	172 ± 9	171 ± 9	173 ± 9	0.587
BMI (kg/m^2^)	28 ± 10	21.3 ± 1.8	35.3 ± 9.5	< 0.001*
Body Fat (%)	33 ± 10	26.2 ± 7.0	39.0 ± 8.2	0.001*
SBP (mmHg)	123 ± 11	115 ± 8	129 ± 8	0.001*
DBP (mmHg)	72 ± 7	68 ± 6	76 ± 7	0.015*
Total cholesterol (mg/dL)	163 ± 26	149 ± 18	177 ± 25	0.007*
LDL (mg/dL)	92 ± 27	76 ± 21	107 ± 23	0.003*
HDL (mg/dL)	52 ± 15	53± 16	50 ± 14	0.644
Triglycerides (mg/dL)	83 ± 59	70 ± 27	96 ± 78	0.301
Glucose (mg/dL)	90 ± 13	83 ± 6	97 ± 14	0.005*
HbA_1c_ (%)	5.4 ± 0.4	5.3 ± 0.3	5.7 ± 0.4	0.030*
Hemoglobin (g/dL)	13.8 ± 1.8	13.7 ± 1.8	14.0 ± 1.8	0.639
Hematocrit (%)	41.0 ± 5.0	40.2 ± 5.3	41.9 ± 4.7	0.453

Note

Values are mean ± SD.

Abbreviations: BMI, body mass index, SBP, systolic blood pressure, DBP, diastolic blood pressure, LDL, low density lipoprotein, HDL, high density lipoprotein.

*p < 0.05.

### 3.2 Endothelin‐1 (ET‐1) response to WBV

**FIGURE 1:**
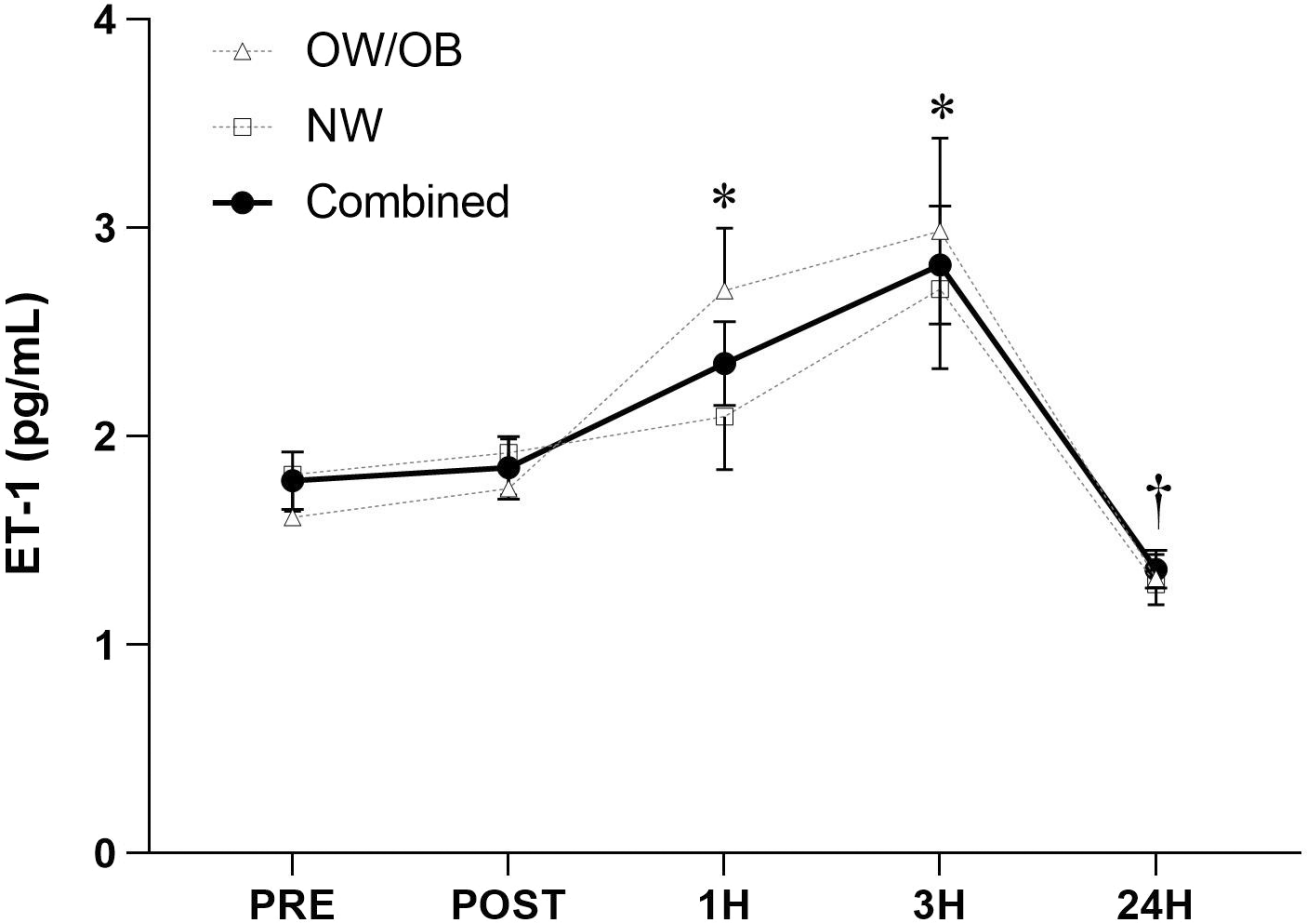
Endothelin‐1 response to whole‐body vibration (WBV) exercise in each group as well as combined. Repeated measures ANOVA. Data presented as mean ± standard deviation (SD). *Significant main effect of time (*p* < 0.05) compared to PRE and POST in the groups combined. †Significant main effect of time (*p* < 0.05) compared to PRE, POST, 1H, and 3H in the groups combined.

Figure 1 illustrates the ET‐1 response in the OB/OW and NW groups as well as in both groups combined. The ET‐1 response to WBV was similar (*F* = 0.180, *p* = 0.677) between groups. However, collapsing across group, a significant main effect of time (*F* = 12.5, *p* < 0.001) was identified. Posthoc analysis for both groups combined identified a significant increase in ET‐1 at 1H (*p* = 0.017) and 3H (*p* = 0.025) compared to PRE. In addition, there was a significant overall decrease in concentrations of ET‐1 at 24H compared to PRE (*p* = 0.019), 1H (*p* < 0.001) and 3H (*p* < 0.001).

### 3.3 Baseline measures of cardiopulmonary and hemodynamic values

Measures of cardiopulmonary gas exchange and hemodynamic values at baseline are presented in Table 2. Relative to body mass, oxygen consumption at baseline was not significantly different between both groups (*p* = 0.637). In addition, when normalized to fat‐free mass, average oxygen consumption remained similar between groups (*p* > 0.05). CTI was greater (*p* = 0.001) in the NW compared to the OW/OB group and EF was higher (*p* = 0.001) in the NW compared to the OW/OB. In addition, EDV was higher (*p* = 0.004) in the OW/OB group compared to NW and there were no significant differences in HR, SV, CO, and VE/VCO_2_ between the two groups.

**TABLE 2:** Baseline cardiopulmonary measures.

Variable	Overall	Normal weight (NW)	Overweight/Obese (OW/OB)	*p*‐value
Heart rate (bpm)	80 ± 16	82 ± 21	79 ± 11	0.693
Stroke volume (mL)	86 ± 20	81 ± 19	90 ± 20	0.296
Cardiac output (L/min)	6.7 ± 1.5	6.4 ± 1.4	7.0 ± 1.7	0.347
Ejection fraction (%)	61 ± 12	69.1 ± 9.5	53.0 ± 8.2	0.001*
End‐diastolic volume (mL)	146 ± 47	117.9 ± 30.0	173.8 ± 44.9	0.004*
Contractility Index	234 ± 102	302 ± 96	166 ± 50	0.001*
VO_2_ (mL/kg/min)	3.3 ± 1.1	3.4 ± 1.3	3.1 ± 0.8	0.637
VO_2_ (mL/kgFFM/min)	5.3 ± 1.8	5.0 ± 2.3	5.6 ± 1.2	0.481

Note

Mean ± SD.

Abbreviations: VO_2_, Oxygen consumption.

*p < 0.05; Independent samples *t*‐test.

### 3.4 Cardiopulmonary and hemodynamic response to WBV

The average cardiopulmonary gas exchange and hemodynamic responses during WBV are presented in Table 3. In response to WBV, CTI was greater (*p* = 0.004) in the NW compared to the OW/OB group. EF during WBV was higher (*p* = 0.001) in the NW compared to the OW/OB; however, EDV was higher (*p* = 0.003) in the OW/OB group compared to NW. The average relative VO_2_ to WBV when normalized to body mass tended to be higher in NW compared to OW/OB (*p* = 0.091); however, VO_2_ normalized to fat‐free mass was similar between groups. There were no significant differences in HR, SV, CO, and VE/VCO_2_ during WBV between the two groups.

**TABLE 3:** Average cardiopulmonary response to WBV

Variable	Overall	Normal Weight (NW)	Overweight/Obese (OW/OB)	*p*‐value
Heart rate (bpm)	101 ± 21	101 ± 26	101 ± 15	0.996
Stroke volume (mL)	105 ± 21	101 ± 20	110 ±22	0.410
Cardiac output (L/min)	10.4 ± 2.1	9.9 ± 1.8	10.9 ± 2.4	0.296
Ejection fraction (%)	61.9 ± 13.1	70.5 ± 11.2	53.3 ± 8.5	0.001*
End‐diastolic volume (mL)	181 ± 64	151 ± 51	211 ± 63	0.030*
Contractility Index	267 ± 126	345 ± 125	193 ± 71	0.004*
VO_2_ (mL/kg/min)	6.8 ± 1.7	7.4 ± 1.6	6.1 ± 1.5	0.091
VO_2_ (mL/kgFFM/min)	11.1 ± 3.1	11.1 ± 3.2	11.2 ± 3.0	0.995
VE/VCO_2_ slope	29.1 ± 3.4	29.1 ± 3.2	29.2 ± 3.8	0.982

Note

Mean ± SD.

Abbreviations: VO_2_, oxygen consumption.

* *p* < 0.05; Independent samples *t*‐test.

## 4 DISCUSSION

Traditional aerobic exercises (i.e., cycling, walking, running) performed regularly reduces the concentration of endothelin‐1 (ET‐1) thereby, reducing the risk of developing cardiovascular diseases (Dow et al., 2017). Whether or not whole‐body vibration (WBV) produces a similar physiological response had yet to be investigated, until now. Accordingly, the purpose of this study was to investigate the effect of acute WBV on circulating ET‐1 over time and determine if adiposity plays a role in the response. Findings of the present study demonstrate that there is an acute increase in circulating ET‐1 at 1H and 3H after acute WBV exercise. In contrast, and perhaps most relevant, there was a decline in plasma ET‐1 24H following WBV. The ET‐1 response to WBV was similar between normal‐weight and overweight/obese participants, and this response was similar to those reported in previous studies following traditional modes of moderate‐intensity exercises.

Elevated basal concentrations of ET‐1 are associated with hypertension and an increased risk of CVD. Perhaps unsurprisingly, traditional modes of exercise training can reduce circulating concentrations of ET‐1 (Dow et al., 2017; Maeda et al., 2001; Maeda et al., 2009), a reduction that can even persist after 4 weeks of exercise cessation (Maeda et al., 2003). The present investigation is the first; however, to examine the acute time course of the ET‐1 response following WBV in normal weight and overweight/obese participants. In the present investigation, there was not only an acute increase in circulating ET‐1 at 1H and 3H after WBV, circulating concentrations of ET‐1 were significantly reduced 24H following WBV. The findings of the current investigation are in agreement with a previous investigation which demonstrated an acute increase in ET‐1 following treadmill running at 55% VO_2_max (McClean et al., 2015). Acute exercise elicits an increase in reactive oxygen species (ROS) and accumulation of free radical species which can disrupt the bioavailability of NO and contribute to an increase in ET‐1 (McClean et al., 2015). In contrast, the reduction in ET‐1 following 24H of WBV could also indicate an improvement in functional ET_B_ receptors, as they are the endothelin receptor primarily responsible for the clearance of ET‐1 (Derella et al., 2022; Fukuroda et al., 1994). In addition, reduction of ET‐1 activation by ET_A_ receptor blockade contributes to an increase in vasodilation in overweight/obese compared to normal weight individuals (Weil et al., 2011). Taken together, these findings highlight the negative role that ET‐1 plays on the impairment of endothelial function in overweight/obese individuals. Nonetheless, findings of the present investigation provide the foundation to support the use of WBV as a novel modality of exercise to reduce circulating concentrations of ET‐1 and reduce CVD risk.

Although the ET‐1 response to exercise is thought to mediate changes in muscle/organ blood flow, peak concentrations of ET‐1 in the present study did not occur until 1 and 3 h post‐WBV. This observation, however, is not completely surprising as studies have shown that although ET‐1 is produced by the vascular endothelial cell, the release of ET‐1 is delayed for ≥30 min after the exercise stimulus (Masaki et al., 1991) (Maeda et al., 1997). The 3H peak in circulating ET‐1 also follows the same pattern that has previously been observed in interleukin‐6 and neutrophils following WBV in humans (Blanks et al., 2020). Most importantly, the increase in ET‐1 in the present study was acute and was followed by a significant reduction 24H following WBV. In contrast to our original hypothesis, this response was similar in both the NW and OW/OB groups, suggesting that routine, chronic adherence to WBV may result in a decrease in circulating ET‐1, independent of adiposity. Future studies are certainly warranted to establish the long‐term effects of WBV on circulating concentrations of ET‐1 and the subsequent reduction in CVD risk.

An increase in cardiopulmonary parameters such as oxygen consumption and cardiac output is key to achieving the cardioprotective benefits of exercise training (Agarwal, 2012). At baseline, the volume of oxygen consumed (VO_2_) was similar between groups. However, in response to WBV, the oxygen consumption tended to be greater in the NW when compared to the OW/OB group, albeit a greater cardiac output and stroke volume in the OW/OB group. In addition, data support that increased adiposity can slow the dynamic response of VO_2_ during an acute cycling exercise (Green et al., 2018). However, the VO_2_ response during WBV observed in the present study is similar to that reported during traditional incremental treadmill exercise (Bk et al., 2019). Although the current investigation examined an acute response to WBV, investigations into how repeated, chronic bouts of WBV exercise affect oxygen consumption are certainly warranted.

In conclusion, despite differences in the cardiopulmonary response between overweight/obese and normal‐weight individuals, the ET‐1 response to WBV was similar between groups. Nonetheless, this is the first investigation in humans to demonstrate that a single bout of WBV exercise elicits a decrease in circulating concentrations of ET‐1 24H later. The post‐acute reduction in ET‐1 24H following WBV suggests that chronic WBV may represent an alternative mode of exercise that can potentially reduce CVD risk in both normal weight and overweight/obese individuals. Future studies are needed to explore the longitudinal effects of WBV on circulating concentrations of ET‐1. In addition, investigations that ascertain the chronic effects of WBV on circulating concentrations of ET‐1 and complementary biomarkers, like nitric oxide, are certainly warranted.

## AUTHOR CONTRIBUTIONS

Adeola A. Sanni‐Ajibaye – Data Acquisition, Data analysis and interpretation, Statistical analysis, Manuscript write up, Manuscript review/revise, Approved the final version. Anson M. Blanks ‐ Study design, Data collection, and analysis, Manuscript draft & revise, Approved the final version. Cassandra C. Derella ‐ Data Acquisition, Manuscript review/revise, Approved the final version. Abigayle B. Simon ‐ Data Analysis, Manuscript review/revise, Approved the final version. Paula Rodriguez‐Miguelez ‐ Data Acquisition, Manuscript review, Approved the final version. Jacob Looney ‐ Data Acquisition, Manuscript review, Approved the final version. Jinhee Jeong ‐ Data Acquisition, Manuscript review, Approved the final version. Jeffrey Thomas ‐ Data Acquisition, data analysis, Manuscript review, Approved the final version. David W. Stepp ‐ Study conception and design, Manuscript review, Approved the final version. Neal L. Weintraub ‐ Data Acquisition, Manuscript review, Approved the final version. Xiaoling Wang ‐ Study conception and design, Manuscript review/revise, Approved the final version. Ryan A. Harris ‐ Data Acquisition, Study conception and design, Manuscript review/revise, Approved the final version.

## CONFLICT OF INTEREST

Authors have no conflicting interest to disclose.
